# Save the pig tail

**DOI:** 10.1186/2055-5660-1-2

**Published:** 2015-04-16

**Authors:** Anna Valros, Mari Heinonen

**Affiliations:** 3 Department of Production Animal Medicine & Research Centre for Animal Welfare, University of Helsinki, Faculty of Veterinary Medicine, P.O. Box 57, 00014 Helsinki, Finland; 4 Department of Production Animal Medicine, University of Helsinki, Faculty of Veterinary Medicine, Paroninkuja 20, 04920 Saarentaus, Finland

**Keywords:** Pig, Tail biting, Tail docking, Risk factors

## Abstract

Tail biting is a common problem in modern pig production and has a negative impact on both animal welfare and economic result of the farm. Tail biting risk is increased by management and housing practices that fail to meet the basic needs of pigs. Tail docking is commonly used to reduce the risk of tail biting, but tail docking in itself is a welfare problem, as it causes pain to the pigs, and facilitates suboptimal production methods from a welfare point-of-view. When evaluating the cost and benefit of tail docking, it is important to consider negative impacts of both tail docking and tail biting. It is also essential to realize that even though 100% of the pigs are normally docked, only a minority will end up bitten, even in the worst case. In addition, data suggests that tail biting can be managed to an acceptable level even without tail docking, by correcting the production system to better meet the basic needs of the pigs.

## Introduction

Tail biting, which is an abnormal behaviour in the domestic pig, is a common problem within the pig industry worldwide. Tail biting has also been identified by farmers as the one of the main welfare problems in pig production [1]. During a tail biting outbreak, pigs bite each other’s tails, causing an increased risk for infection and carcass condemnations [2, 3]. Tail biting is thus a serious welfare and economical issue, and it is of great importance to minimize the problem.

Tail docking is commonly used as a measure to prevent tail biting. Tail docking includes the amputation of a part of the tail [4]. However, tail docking does not solve the problem of tail biting totally, and also in itself induces pain to the docked pigs [5]. In addition, as suboptimal housing and management are well known risk factors for tail biting [6], docking the tail only serves to alleviate the symptom of reduced welfare. Thus, tail docking facilitates a production method where the underlying problem itself can be partly ignored and pigs can be managed in environment taking less in consideration their real needs.

Tail docking is basically banned in the EU (Council Directive 2008/120/EC, Annex 1). However, the regulation allows tail docking, if no other methods have been successful in reducing the tail biting incidence satisfactorily. This has led to tail docking being very common throughout EU, with a total ban being enforced only in a few member countries: Finland, Sweden and Lithuania [6]. In most EU countries, approximately 99% of pigs are docked [6]. Recently, there has been a lot of political discussion on the topic, and suggestions on possibly enforcing the ban more strictly have been made. However, if tail docking is to be totally banned changes will have to be made both in the attitudes and the practises of the pig industry. To address this, the aim of this review is to examine the costs and benefits of a more strictly enforced tail docking ban, as well as to shortly discuss possibilities for managing tail biting on-farm.

## Review

### Prevalence of tail biting and efficiency of tail docking

When comparing prevalence of tail biting from existing literature, it is important to consider the great variation in the sampling methods and the definition of tail biting cases. Special care needs to be taken when comparing data from routine abattoir data and data recorded separately by researchers on-site. Keeling et al. [7] reported that while the researchers scored approx. 7% of pig tails as injured or shortened, using the criteria used by the same slaughterhouses (half of or less of the tail left) would have resulted in a prevalence below 2% in the same data of 15,068 slaughtered pigs.

In scientific studies, the prevalence of serious tail biting, including fresh signs of tissue damage, varies greatly from study to study, with undocked pigs having a prevalence between 2 and 12% [8, 2] docked pigs up to about 3% [8, 9]. Hunter et al. [10] showed that tail docked pigs in a dataset of 27,870 pigs from 450 farms, recorded at six UK abattoirs, had an overall prevalence of tail biting of 2.4%, as compared to 8.5% in undocked pigs. Harley et al. [11] reported that in a data of 36,963 pigs, where over 99% were tail docked, as many as 58.1 % had detectable signs of tail lesions, while 1% had severe lesions. Thodberg et al. [12] found, in an experimental study on four different farms, that the risk of tail biting was only significantly reduced when at least ¾ of the tail was docked. The risk of tail biting was 4.6 - fold in pigs with intact tails as compared to pigs with only ¼ tail left, while the risk was 2.8 and 3.3 – fold, respectively, for pigs with ¾ or ½ of the tail left at docking. In conclusion, tail docking reduces tail biting, but there is some variation. One can estimate that docking reduces the prevalence for tail biting 2-4-fold, but that there are very few studies that have focused on this question without being biased for recording methods and management of the pigs.

### Negative impact of tail biting

In addition to the acute pain and lowered welfare for the victim, biting causes a significantly increased risk of infections. Heinonen et al. [3] showed that acute phase proteins (C-reactive protein, Serum Amyloid A and Haptoglobin) were significantly increased in tail biting victims, in comparison to healthy controls, and that the more severe the tail biting was, the larger the acute phase protein reaction. Furthermore it has been shown that tail biting victims have an increased incidence of abscesses and arthritis at slaughter, which leads to carcass condemnations [2, 13].

Tail biting also causes stress-related changes in the victim pigs: Valros et al. [14] showed that chronically bitten pigs had a lower cortisol reaction to acute stress than that of non-bitten pigs, indicating hypocortisolism, possibly induced by the chronic stress caused by being bitten, or by the increased infection status caused by chronic tail biting [14]. An increased stress level in victim pigs during a recent outbreak of tail biting was also shown by Munsterhjelm et al. [15], indicated by an increase in adrenal total and cortical area, increased evening cortisol level and T3 suppression.

Tail biting victims have reduced weight gain and carcass weight [13, 14, 16]. This might be due to the increased infection pressure or the stress itself, but it could also be caused by changes in feeding patterns [17].

As tail biting leads to an increased risk of infection and other health problems [2, 3, 18], bitten pigs should be treated with antibiotics to avoid future problems. Tail biting thus increases medical costs [19] and labour demands due to medication. In addition, tail biting increases the risk for other health problems, such as locomotion disorders [13, 20].

We have not been able to identify a good data set for estimating the on-farm mortality caused by tail biting, but anecdotal reports indicate this can be an important cause of losses too, especially when severe outbreaks occur. However, taking the above negative effects together, tail biting can lead to severe animal welfare problems, as well as to economic losses.

### Negative impact of tail docking

Even though tail docking is a method to reduce tail biting, tail docking in itself is a welfare issue. Tail docking causes acute pain and stress, as indicated by both behavioural and physiological changes [5, 21, 22] and the method used does not influence this very much [4, 6]. Studies have also failed to report efficient methods of pain alleviation during tail docking [5].

Regarding the effect of tail docking on piglet growth, studies have given contradictory results: Marchant-Forde et al. [21] reported a reduction in growth rate of tail docked pigs up to 14 d, when using a hot cautery docking as compared to blunt-cut and sham-cut treatments. Also Zhou et al. [23] showed that teeth clipping and tail docking reduced weight gain for as long as until 70 d of age. On the other hand, Sutherland et al. [22] found a better growth rate in docked than undocked pigs at 7 weeks of age. However, in contrary to the pigs in the study by Marchant-Forde et al. [21], these undocked pigs also suffered from more tail lesions than the docked ones, which could explain the reduced weight gain compared to docked pigs.

The long-term pain associated with tail docking is still not fully understood [4]. One suggested mechanism of why tail docking reduces tail biting is that tail docking causes neuroma formation in the tail tip [24], which in itself makes the tail more sensitive, and thus might increases avoidance behaviour of pigs when being bitten. Herskin et al. [25] confirmed that tail docking does cause an increased prevalence of neuroma formation, and that the bigger the docked part of the tail is, the higher the prevalence of neuroma formation. Neuromas are known to increase the risk for spontaneous pain and hypersensitivity [26] and thus may indicate prolonged pain experience due to tail docking, as well as an increased pain perception if the tail is later on bitten.

As tail docking leads to major tissue damage, there is an evident risk of infection. Even though the risk of infection due to tail docking is probably much smaller than that caused by severe biting, also unhygienic tail docking has been suggested to be a potential risk for eg. spinal abscesses [27] and arthritis [28].

A dataset collected at one big abattoir in Finland in 2000, including 10,852 pigs from 479 farms showed that even in cases of healed tail damage, there was a significantly increased prevalence of arthritis and abscesses in the carcasses compared to healthy-tailed pigs [2]. Although the study did not record docking separately, the authors do state that a big part of the 2476 pigs with healed tail damage most probably included docked pigs, because tail docking was not forbidden in the country at that time. This data is only speculative in indicating a long-term negative effect on pig health and carcass quality of tail docking. There is, however, not very much scientific or epidemiological data available, and further studies are warranted.

### Risk factors for tail biting

There are several reviews covering the risk factors for tail biting [29, 30], highlighting e.g. the lack of manipulable material, poor climate, feeding problems, dysfunctional social structure and poor pen layout Here we will concentrate on only giving some more details on certain factors that have received more attention during the recent years.

#### Risk factors at individual pig level

Recent studies have shown that there are several phenotypic differences between pigs from different categories of tail biting-related behaviour in both behavioural [31–33] (autonomic regulation [32], stress level [15] and neurotransmission [34]. These differences suggest underlying traits that could influence the ability of the animals to meet environmental challenges.

The above mentioned studies of pigs of different phenotypes of tail biting have, however been performed on pigs already observed to bite or become bitten, which makes it difficult to separate cause and effect. Zonderland et al. [35], showed that prior to a tail biting outbreak occurring, tail biters receive more aggression and are chased more often than control pigs. Future biters also manipulated enrichment more frequently and tended to sit and kneel more prior to the outbreak, which might be indicative of a higher level of stress.

It has long been well-known that there is an effect of gender on the risk of becoming a tail biting victim [2, 36, 37]. Zonderland et al. [38] showed that females are more prone to bite. However, this gender effect is not straightforward, but can probably be influenced by the way animals of different genders are mixed. Interestingly, Sinisalo et al. [16] did not find an effect of gender on the risk of being tail bitten in a sample of 3190 pigs, including also boars, on a farm where animals were mainly housed in single-sex groups.

There are indications of a possibility for genetic development of pigs with a lower risk of tail biting as the risk of being tail bitten is influenced by breed [16, 39]. Tail biting is probably heritable, and genetically connected to a high lean tissue level and low back fat level, both characteristics may be favoured by modern selection [40]. More recent studies have shown that pigs that stay neutral, ie. are neither bitten or performing biting in pens where tail biting do occur, differ significantly in their gene expression from other phenotypes. Interestingly one of these genes was related to leanness, suggesting that neutral pigs are fatter than the other phenotypes [33].

#### Health as a risk factor

Suboptimal health as a risk factor for tail biting has not received very much attention so far. In an epidemiological case–control study, Moinard et al. [41] showed that the presence of respiratory diseases and a high post-weaning mortality on the farm increased the risk of tail biting. Marques et al. [13] showed that tail biting lesions were connected to locomotory problems. Similar findings were reported by Niemi et al. [20], also showing that lame pigs actually run a greater risk of becoming victims of tail biting. Munsterhjelm et al. [18] reported also an increased prevalence of respiratory infections in post mortem examinations of acutely bitten pigs. As tail biting occurred only a few days before the pigs were euthanized, it is not possible to estimate which was the cause and effect, the respiratory problems or the tail biting.

#### Manipulable material

The lack of manipulable material is probably the most significant risk factor for tail biting [30]. Adding manipulable material per se is, however, not a guarantee for efficient prevention of tail biting. The material used needs to be designed and chosen to fit the behavioural needs of pigs [30]. Studies indicate that straw is more efficient in reducing the risk for tail biting than eg. point-source objects [30]. However, using satisfactory amounts of straw is not always feasible. Some kind of a solid manipulable object can be efficient as well: Telkänranta et al. [42] reported that fresh wood was more efficient than a branched chain or plastic tubes in reducing tail biting.

Recent studies underline that the early rearing period of pigs is important for the development of tail biting risk. Munsterhjelm et al. [43] showed that pigs that had bedding during the first 4 weeks of life had a lower prevalence of harmful social behaviour (including tail biting) when in the fattening unit. In the study by Telkänranta et al. [44] the severity of tail biting after weaning could be reduced by providing nursing piglets with sisal ropes and newspaper.

#### Feeding-related risk factors

Competition for feed has been identified as a risk factor [41]. Individual feeders caused a substantial increase in tail bites in the area close to the feeder, as compared to other parts of the pen [45]. One way for an individual pig to avoid getting tail bitten might actually be to reduce feeding. Palander et al. [46] showed that pigs that remained non-bitten non-biters in tail biting pens had changes in their intestinal morphology, indicative of some level of anorexia. Wallenbeck and Keeling [47] further showed that pigs that were to become victims had a greater frequency of feeder visits than other pigs already 2–5 weeks before the start of tail biting in the pen. This might make them especially at risk to becoming victims of tail biting.

### Intervention

Tail biting behaves like an epidemic: Single cases of tail biting can develop into serious outbreaks [48], and the second case typically occurs quickly after the first one [20], which underlines the importance of quick intervention. However, it needs to be remembered that outbreaks differ in the way they develop [48] and that tail biting have different motivational background and aetiology [49].

Functional curative measures include adding more straw [50] and removing the biter [19, 50]. To be able to intervene at an early stage, it is important to identify an outbreak early. In addition to signs of actual biting, activity level and restlessness, as well as tail posture are good indicators of a possible tail biting episode in the pen. Prior to an outbreak, pigs can be seen to keep their tails in a low posture, tucked between their legs [48, 51]. Another promising method for early identification of tail biting is following changes in feeding behaviour. Wallenbeck and Keeling [47] reported that the frequency of visits to automatic feeders decrease in tail biting pens already weeks before the outbreak and Viitasaari et al. [17] showed a change in feeding behaviour when tail biting started.

### Management decisions to reduce tail biting risk

Using straw is one way to handle tail biting and manage it at acceptable level [52]. Hunter et al. [10] showed that farms providing straw and natural light had a highly reduced level of tail biting in both docked (1.2%) and undocked pigs (4.3%) as compared to an overall level of 2.4 vs 8.5%. They also showed that providing proper feeding space reduced the prevalence to 3.9% in undocked pigs.

Even though abattoir data probably underestimates the total tail biting occurrence, the situation appears promising when considering countries where tail docking is prohibited. In Finland, where tail docking is totally prohibited, but the relevant legislation has otherwise been similar to the rest of the EU, the prevalence of tail biting, based on abattoir data from the two biggest slaughterhouses in 2013, was 2,3% (data from approximately 1,6 million slaughter pigs) (Jukola and Tirkkonen, personal communication). These pigs are typically housed at a density of 0.8-0.9 m^2^/pig, some manipulable material is given daily and pigs are normally ensured enough feeding space by using trough feeding. They also they have continuous free access to water. Thus, even though the practice in Finland is not very different from countries where docking is used, some of the pigs’ basic needs are taken into account to a higher degree than the EU-legislation requires. In Sweden, a data set of approximately 15,000 pigs at two slaughterhouses showed that 1,5% vs 1,9% of the pigs had half or less of their tail left [7]. Using international Welfare Quality^®^ data, a recent EFSA report [30] concludes that tail biting can probably be managed with proper housing and management, without increasing the risk for tail biting.

There is a huge variation in tail biting prevalence between farms, for example between 0 and 50% fresh tail injuries in the 497 batches recorded by Valros et al. [2]. However, there seems to be no pig production system that totally protects against tail biting: the problem occurs at some level in most conventional farms, with 95% of the batches included in the study by Valros et al. [2] having at least one case of fresh tail biting. Tail biting also occurs in organic farming [53] and in outdoor production [54]. It is therefore not realistic to expect tail biting to not occur at all, but we do claim that an acceptable and manageable level can be reached.

### Cost-benefit model

**Figure 1:**
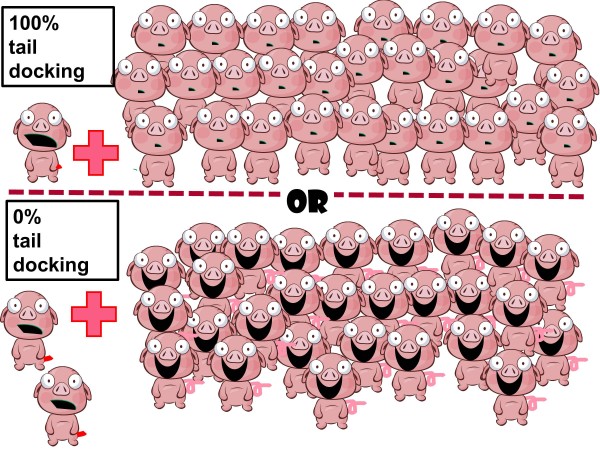
**Theoretical illustration of the result of a 100% vs a 0% tail docking policy.** Tail docking causes some pain to 100% of the pigs, and reduces the risk of tail biting (approximately 2-fold, based on available abattoir data). Even though the risk for tail biting might be higher if tails are not docked, and the pain caused by biting more intense than the pain caused by docking, the non-bitten pigs are fully spared the pain due to tail docking in a 0% docking scenario. Pigs that are both docked and bitten suffer the most pain. In addition to this, undocked pigs will likely be kept on better management level.

Until now, the tail biting/tail docking discussion has mainly focused on the negative effects of tail biting, and on the other hand, on the positive effect of tail docking in reducing tail biting risk. However, to make a more balanced estimation of the overall effect of a possible strict enforcement of the tail docking ban within EU, it is important to consider all the elements of the dilemma. To make this more concrete, we propose the equation below. One of the most central calculations within the analyses, ie the balance between 100% tail docking with somewhat reduced biting risk versus no tail docking and slightly higher level of tail biting is illustrated further in Figure 1. The assumptions below are based on an expected level of tail biting (at the level seen in abattoir data) of 1% in docked pigs and 2% in non-docked pigs. This assumption, however, needs to be adjusted when tail biting management is improved also in non-docked pigs.

The suggested equation model is based on two theoretical scenarios: 100% tail docking versus 0% tail docking.

The aim of this paper was not to estimate monetary costs as such, thus we have not attempted to add any sums into this calculation. ‘Cost’ instead refers more generally to adverse effects, and includes both animal welfare and economic considerations.



Where COST OF TAIL DOCKING includes the following elements: 100% * (TDpain + TDInf + TDworkload); COST OF TAIL BITING includes the following elements: 1% * ((TBpain*TD) + TBinf + TBincome_reduction + TBmedication + TBworkload)



Where COST OF TAIL BITING includes the same elements as above, but as at a higher level, ie.: 2% * (TBpain + TBinf + TBincome_reduction + TBworkload); and BENEFITS OF NO TAIL DOCKING includes 100% * pigs experiencing improvements in management and housing + x * better image of pig production and COST OF TAIL BITING PREVENTION includes the cost of measures taken on-farm to reduce the risk of tail biting, such as reduced animal density, increased use of manipulable material and increased feeding space.

*TDpain (= pain experienced due to tail docking) is assumed to be < than TBpain (= pain due to tail biting)*.

*TDinf (=risk of infections due to tail docking) is assumed to be < than TBinf (= risk of infection due to tail biting).*

*TDworkload (= workload caused by tail docking of individual pigs) is assumed to be < than TB workload (= workload due to medication and handling of individual tail biting victims and tail biters)*.

TBpain*TD is based on the assumption that tail biting is more painful in docked pigs than in non-docked.

*TBincome_reduction refers to costs of tail biting due to weight reduction, mortality and carcass condemnations*.

## Discussion and conclusions

Tail biting is a serious problem, both from an animal welfare and an economical point of view. However, even though tail docking can reduce the risk of tail biting, the negative consequences of tail docking cannot be ignored. In addition, available data indicate that tail biting can be managed to an acceptable level, maybe even to a comparable level, even when tail docking is not used. Some investment in improving management and housing is needed, but there does not appear to be a need to change the production system totally, such as converting to deep bedded or free-range systems.

We do not claim that the suggested equations for the cost-benefit analyses are complete, but suggest that this might be used as a starting point for further discussion. It also gives indication of the areas where there is a need for further data collection in order to ensure a holistic approach to the dilemma in question. For some elements of the equation useful data already exists, such as on the effect of tail biting on carcass condemnation prevalence, and on the acute pain caused by tail docking. Other parts still need further data collection, such as the level of pain caused by tail biting and the long-term effects of tail docking. Furthermore, some elements are more difficult than others to fill in with quantitative data, for example the effect of tail docking and tail biting on the image of the EU pig production. Although recognising these weaknesses, we believe that a more systematic and holistic approach would benefit the discussion and make future decisions more objective. A central point is to recognise that even though there is no proof that tail biting cannot be avoided totally, tail docking is usually performed on 100% of the pigs at a farm level, while tail biting only occurs in a small proportion of animals.

Farmers in NL indicate that they are afraid of high incidences of tail biting to occur if they do not dock [1]: this attitude makes it difficult to enforce a total ban on docking. To overcome the barrier, farmers need to be convinced that tail biting is manageable even without tail docking. However, this does mean adapting somewhat different farming practises. Farmers should also not expect that tail biting would disappear totally. It needs to be accepted that a non-docking policy might increase the average risk for tail biting, at least before new management practises are successfully adopted, even though an uncontrollable increase is not to be expected.
